# Antioxidant, 5-Lipoxygenase Inhibitory and Cytotoxic Activities of Compounds Isolated from the *Ferula lutea* Flowers

**DOI:** 10.3390/molecules191016959

**Published:** 2014-10-22

**Authors:** Mansour Znati, Hichem Ben Jannet, Sylvie Cazaux, Jean Pierre Souchard, Féthia Harzallah Skhiri, Jalloul Bouajila

**Affiliations:** 1 Université de Toulouse, Faculté de Pharmacie de Toulouse, Laboratoire des IMRCP UMR CNRS 5623, Université Paul-Sabatier, 118 route de Narbonne, F-31062 Toulouse, France; E-Mails: Znatimansour@yahoo.fr (M.Z.); sylvie.cazaux2@univ-tlse3.fr (S.C.); souchard@chimie.ups-tlse.fr (J.P.S.); 2 Laboratoire de Chimie Hétérocyclique, Produits Naturels et Réactivité (CHPNR), Equipe Chimie Médicinale et Produits Naturels, Département de Chimie, Faculté des Sciences de Monastir, Université de Monastir, Avenue de l’Environnement, 5019 Monastir, Tunisia; 3 Laboratory of Genetic, Biodiversity and Valorization of Bioresources, Higher Institute of Biotechnology of Monastir, University of Monastir, 5019 Monastir, Tunisia; E-Mail: fethiaprosopis@yahoo.fr

**Keywords:** *Ferula lutea*, secondary metabolites, phenolic, antioxidant, anti-inflammatory, cytotoxic

## Abstract

A phytochemical investigation of the *Ferula lutea* (Poir.) Maire flowers has led to the isolation of a new compound, (*E*)-5-ethylidenefuran-2(5H)-one-5-*O*-β-d-glucopyranoside (**1**), designated ferunide, 4-hydroxy-3-methylbut-2-enoic acid (**2**), reported for the first time as a natural product, together with nine known compounds, verbenone-5-*O*-β-d-glucopyranoside (**3**), 5-*O*-caffeoylquinic acid (**4**), methyl caffeate (**5**), methyl 3,5-*O*-dicaffeoylquinate (**6**), 3,5-*O*-dicaffeoylquinic acid (**7**), isorhamnetin-3-*O*-α-l-rhamnopyranosyl(1→6)-β-d-glucopyranoside, narcissin (**8**), (−)-marmesin (**9**), isoimperatorin (**10**) and 2,3,6-trimethylbenzaldehyde (**11**). Compounds **3**–**10** were identified for the first time in *Ferula* genus. Their structures were elucidated by spectroscopic methods, including 1D and 2D NMR experiments, mass spectroscopy and X-ray diffraction analysis (compound **2**), as well as by comparison with literature data. The antioxidant, anti-inflammatory and cytotoxic activities of isolated compounds were evaluated. Results showed that compound 7 exhibited the highest antioxidant activity with IC_50_ values of 18 ± 0.5 µmol/L and 19.7 ± 0.7 µmol/L by DPPH radical and ABTS radical cation, respectively. The compound **6** exhibited the highest anti-inflammatory activity with an IC_50_ value of 5.3 ± 0.1 µmol/L against 5-lipoxygenase. In addition, compound **5** was found to be the most cytotoxic, with IC_50_ values of 22.5 ± 2.4 µmol/L, 17.8 ± 1.1 µmol/L and 25 ± 1.1 µmol/L against the HCT-116, IGROV-1 and OVCAR-3 cell lines, respectively.

## 1. Introduction

Plants are still used as a source of large-scale original and novel chemical structures. New compounds serve as an index to promote new medicines herbal and dietary supplements. Phenolic substances, which are largely found in most plants, exhibit a wide range of biological effects including anti-inflammatory, antimicrobial and anticancer effects [1] Secondary metabolites such as alkaloids, flavonoids, tannins, saponins, generally produced by plants for their defense mechanisms, have been implicated in the therapeutic properties of most medicinal plants [2].

The *Ferula* genus includes 170 species [3]. Just four species of *Ferula* were found in Tunisia (*F. communis*, *F. lutea*, *F.tunetan a* and *F. tingitana*) [4]. Some varieties are very advantageous to humankind since they are used in regular ordinary nutrition and traditional medicine. According to our own survey in different regions in Tunisia, it has been indicated that some of these species are also useful against delirium and convulsion, activities probably associated with the presence of antioxidants [5]. A successful chemical survey of the roots of the *F. lutea* has contributed to the isolation of new dihydrofuranocoumarins, together with eight known compounds [6]. It is well known that the *Ferula* genus has a variety of coumarins [7], phenolics such as chlorogenic acid, gallic acid and pyrogallol [8]. Moreover, the *Ferula* genus is referred to be a good source of biologically active compounds like sesquiterpene derivatives [9,10], daucanes [11], germacranes [9] and sesquiterpene coumarins identified in the roots of the plants. Therefore, theroots are a better source for isolating sesquiterpene coumarins than the aerial parts [12,13].

Here, we report on the isolation and structural elucidation of six compounds from the *n*-butanol extract of the flowers of *F. lutea*: two new compounds: ethylidenefuran(*E*)-5-ethylidenefuran-2(5*H*)-one-5-*O*-β-d-glucopyranoside (**1**),named ferunide, and4-hydroxy-3-methylbut-2-enoic acid (**2**), and three known compounds:verbenone-5-*O*-β-d-glucopyranoside (**3**), chlorogenic acid (**4**), methyl caffeate (**5**). Six known compounds were also isolated from the ethyl acetate extract of the flowers of *F. lutea*: methyl 3,5-*O*-dicaffeoylquinate (**6**), 3,5-*O*-dicaffeoylquinic acid (**7**), narcissin isorhamnetin-3-*O*-α-l-rhamnopyranosyl(1→6)-β-d-glucopyranoside (**8**), (−)-marmesin (**9**), isoimperatorin (**10**) and 2,3,6-trimethylbenzaldehyde (**11**). Compounds **3-10** were not previously identified in the *Ferula* genus.The antioxidant potential of the isolated compounds was evaluated by DPPH^●^ and ABTS^●+^ assays. The anti-inflammatory effect was also examined via 5-lipoxygenase inhibitory activity. Finally, the cytotoxic activity was evaluated using the MTT assay on the HCT-116 human colon cancer cell line and IGROV-1, OVCAR-3 human ovary cells lines.

## 2. Results and Discussion

### 2.1. Structure Determination

The*n*-butanol and ethyl acetate extracts of the flowers of *F. lutea* were fractionated by successive column chromatography to afford two new compounds **1** and **2**, together with eight known compounds **3**–**10** not previously identified in the *Ferula*genus (Table 1).

**Table 1:** Structures of compounds **1**–**11** isolated of *F. lutea* flowers.

Compound	Name	Structure
**1**	(*E*)-5-Ethylidenefuran-2(5*H*)-one-5-*O*-β-d-glucopyranoside	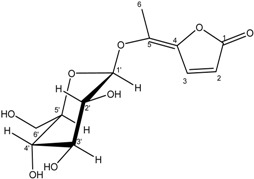
**2**	4-Hydroxy-3-methylbut-2-enoic acid	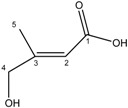
**3**	Verbenone-5-*O*-β-d-glucopyranoside	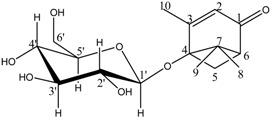
**4**	5-*O*-Caffeoylquinic acid	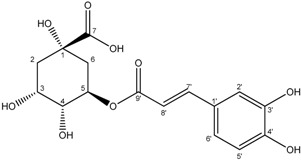
**5**	Methyl caffeate	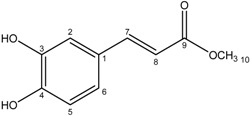
**6**	Methyl 3,5-*O*-Dicaffeoylquinate	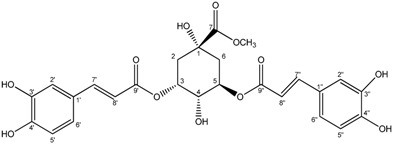
**7**	3,5-*O*-Dicaffeoylquinic acid	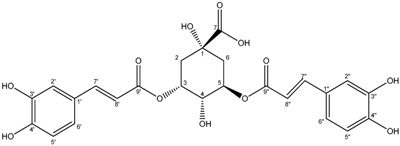
**8**	Isorhamnetin-3-*O*-α-l-rhamnopyranosyl(1→6)-β-d-glucopyranoside,narcissin	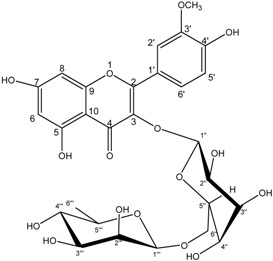
**9**	(−)-Marmesin	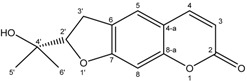
**10**	Isoimperatorin	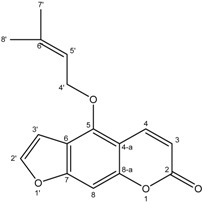
**11**	2,3,6-Trimethylbenzaldehyde	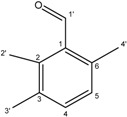

Compound **1** was isolated as a white amorphous powder. Positive DCI-HRMS of this substance gave pseudo-molecular ion peaks [M+H]^+^ at *m/z* 289.0916, which is consistent with the molecular formula C_12_H_16_O_8_ and five degrees of unsaturation. The IR spectrum displayed intense absorption bands at 3341 (OH), 1606 (C=C).The ^1^H and ^13^C-NMR spectral data of compound **1** were assigned in Table 2.

**Table 2:** ^1^H (300 MHz, δ in ppm, *J* in Hz) and ^13^C (75 MHz, δ in ppm) NMR data of compounds **1** and **2**.

Position	1 (in CD_3_OD)		2 (in DMSO-*d*_6_)	
	^1^H	^13^C	^1^H	^13^C
1	-	175.8	-	167.5
2	6.49, d (5.7)	115.9	5.84, m	113.0
3	8.04, d (5.7)	155.7	-	158.2
4	-	163.2	3.91, br s	65.1
5	-	142.2	1.95, d (1.5)	15.1
6	2.44, s	14.3		
1′	4.85, d (7.5)	104.0		
2′	3.20-3.39, m	74.6		
3′		77.1		
4′		69.7		
5′		76.7		
6′a	3.83, dd (12; 2.4)	61.1		
6′b	3.67, dd ( 12; 5.4)			
OH			5.16, s	
OH (acid)			11.86, s	

**Figure 1:**
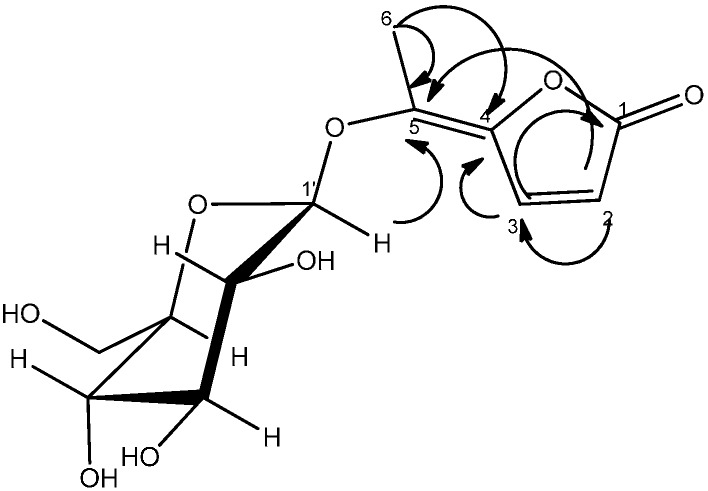
HMBC Correlations of compound **1**.

The ^1^H-NMR spectrum of compound **1** displayed the presence of a methyl group attached to a double bond at δ_H_ 2.44 (s, H-6) and two vicinal methine protons at δ_H_ 8.04 (d, *J = * 5.7 Hz, H-2) and 6.49 (d, *J = * 5.7 Hz, H-3) [14,15]. The presence of the disubstituted γ-methylene-γ-lactone moiety was confirmed by the ^13^C-NMR signals at δ_C_ 175.8 (C-1, CO), 163.2 (C-4, C), 155.7 (C-3, CH), 142.2 (C-5, C) and 115.9 (C-2, CH), and was reinforced by the HMBC spectrum showing the correlation of the methine protons H-2 and H-3 with C-1, C-4 and C-5. The position of the methyl group (δ_H_ 2.44; δ_C_ 14.3) was ascertained by its long range correlation ^1^H-^13^C with the two ethylenic quaternary carbons C-4 and C-5 resonating at δ_C_ 163.2 and 142.2, respectively, indicating that this methyl group was attached to C-5. A careful examination of the ^1^H-^1^H COSY, HSQC and HMBC spectra (Figure 1) indicated additional correlations permitting the establishment of the connectivities.

The presence of the β-D-glucopyranoside system was deduced from the ^13^C-NMR signals at δ_C_ 104.0 (C-1'), 74.6 (C-2'), 77.1 (C-3'), 69.7 (C-4'), 76.7 (C-5') and 61.1 (C-6'). These spectral data were reinforced by the 1H-NMR spectrum exhibiting the typical pattern of β-D-glucopyranoside moiety. This spectrum showed signals at δ_H_ 4.85 (d, *J = * 7.5 Hz, H-1'), 3.83 (dd, *J*_1_ *=* 12, *J*_2_ *=* 2.4 Hz, H-6'a), 3.67 (dd, *J*_1_ *=* 12, *J*_2_ *=* 5.4 Hz, H-6'b) and 3.20–3.39 (m, 4H) attributable to H-2', H-3', H-4' and H-5'.The location of the sugar moiety was evident with the help of the HMBC spectrum showing the correlation between H-1′ and C-5 of the aglycone.

The (*E*) configuration of the double bond (C-4,5) in **1** was proposed from the NOESY spectrum showing the absence of any *nOe* between the ethylenic proton H-3 (δ_H_ 8.04, d, *J = * 5.7 Hz) with the methyl protons H-6 (δ_H_ 2.44,s). From these data, the structure of **1** was identified as (*E*)-5-ethylidenefuran-2(5*H*)-one-5-*O*-β-d-glucopyranoside, and denoted as ferunide.

Compound **2** was obtained as colorless crystals. The negative ES-MS of this substance showed a pseudo-molecular ion peak [M−H]**^− ^** at *m/z* 115.0393 compatible with the molecular formula C_5_H_8_O_3_ (M_w_ = 116) and two degrees of unsaturation. The IR spectrum of compound **2** showed absorption bands typical of a conjugated carbonyl (1699 cm^−1^) and an absorption band at 3333 cm^−1^ characteristic of a hydroxyl group.

The ^1^H-NMR spectrum of compound **2** revealed the presence of methyl group attached to a double bond at δ 1.95 (d, *J = * 1.5 Hz, H-5), an ethylenic methine proton at δ 5.84 (s, m, H-2) and an oxygenated methylene at δ 3.91 (br s, H-4). The ^13^C-NMR showed five carbon resonances, which were identified by HSQC and HMBC experiments as a methyl (δ 15.1, C-5), a methine (δ 113.0, C-2), a methylene (δ 65.1), a carbonyl carbon (δ 167.6, C-1) and a quaternary carbon (δ 158.2, C-3). The sequence of the carbon skeleton of compound **2** was easily deduced from the HMBC spectrum showing the correlations H-2/C-1, H-2/C-3, H-2/C-4, H-2/C-5, H-5/C-3, H-5/C4 and H-4/C-3.

**Figure 2:**
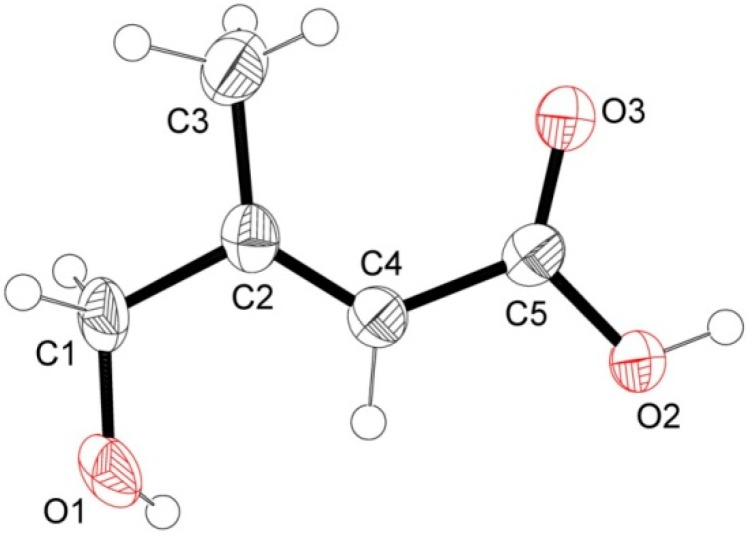
ORTEP drawing of compound **5** showing the atomic numbering scheme. Displacement ellipsoids are plotted at 50% probability level.

White X-ray quality crystals of compound **2** was obtained by crystallization in CHCl_3_/MeOH (95:5). The X-ray diffraction analysis of **2** was carried out on a single crystal (Figure 2). This study confirmed the structure of compound **2** and clearly established that the absolute configuration of the double bond is (*E*).

From these data, the structure of **2** was identified as (*E*)-4-hydroxy-3-methylbut-2-enoic acid, indicated for the first time as a natural compound.

Compounds **3**–**11** were identified as verbenone-5-*O*-β-d-glucopyranoside (**3**), a compound cited only twice in the literature [16,17], chlorogenic acid (**4**) [18], methyl caffeate (**5**) [19], methyl 3,5-*O*-dicaffeoylquinate (**6**) [20], 3,5-*O*-dicaffeoylquinic acid (**7**) [21], isorhamnetin-3-*O*-α-l-rhamno-pyranosyl(1→6)-β-d-glucopyranoside (narcissin, **8**) [22], (−)-marmesin (**9**) [23], isoimperatorin (**10**) [24] and 2,3,6-trimethylbenzaldehyde (**11**) [25] by spectroscopic analyses and comparison with literature data.Compounds **2**–**10** were identified for the first time in the *Ferula* genus, whereas compound **11** was previously identified in the essential oil of *F. lutea* [25].

### 2.2. Antioxidant Activity

In this study, DPPH radical scavenging activity and ABTS radical cation activity were measured to assess the antioxidant activity of compounds from *F. lutea* flowers. The results of the antioxidant activity are presented in Table 3. Only the phenolic compounds were tested of the antioxidant activity.

**Table 3:** Antioxidant (DPPH and ABTS^+.^ assays), 5-lipoxygenase inhibitory and cytotoxic (HCT-116, IGROV-1 and OVCAR-3 cells lines) activities of compounds **1** to **11**.

Compound	IC_50_ (µmol/L) DPPH Assay	IC_50_ (µmol/L) ABTS+ Assay	5-Lipoxygenase Inhibitory		Cytotoxic Activity (IC_50_ µmol/L)		
			(% at 80 µmol/L)	IC_50_ (µmol/L)	HCT-116	IGROV-1	OVCAR-3
**1**	nt	nt	16.9 ± 1.2		>100	>100	>100
**2**	nt	nt	0		>100	>100	>100
**3**	nt	nt	12.4 ± 0.8		>100	>100	>100
**4**	127.4 ± 1.2	132.2 ± 1.5	17.1 ± 1.1		>100	>100	>100
**5**	39.2 ± 0.5	40.8 ± 0.4	0		22.5 ± 2.4	17.8 ± 1.1	25 ± 1.1
**6**	26.6 ± 1.3	20.0 ± 0.3		5.28 ± 0.1	>100	>100	>100
**7**	18.0 ± 0.5	19.7 ± 0.7		10 ± 0.2	>100	>100	>100
**8**	>300	>300	0		>100	>100	>100
**9**	nt	nt	0		50.0 ± 4.9	>100	>100
**10**	nt	nt	0		>100	>100	>100
**11**	nt	nt	0		>100	>100	>100
Vitamin C	25.0 ± 1.1	23.3 ± 0.1	-				
Doxorubicin					0.18 ± 0.01		
Tamoxifen						2.0 ± 0.1	1.3 ± 0.1
NDGA	-	-	98.0 ± 0.1	6.2 ± 0.5			

IC_50_ values represent the mean ± standard deviation of three parallel measurement (*p* < 0.05). nt: not tested.

Among the compounds from *F. lutea* flowers **7**, **6** and **5** exhibited the most potent DPPH free radical scavenging activity, with IC_50_ values of 18.0 ± 0.5 µmol/L, 26.6 ± 1.3 µmol/L and 39.2 ± 0.5 µmol/L, respectively. Compound **4** showed a moderate activity, with an IC_50_ value of 127.4 ± 1.2 µmol/L, compared to the positive control, ascorbic acid (vitamin C) (IC_50_ = 25.0 ± 0.1 µmol/L). The ABTS^+.^ scavenging activity of compounds from *F. lutea* flowers was similar to the DPPH free radical scavenging activity. Although the compound **8** is phenolic, does not have interesting antioxidant activity.

Compound **8** was previously tested by the DPPH radical scavenging activity assay and showed no activity [22].Compound **5** was previously tested for DPPH radical scavenging activity with an IC_50_ = 14.0 ± 2.3 µmol/L [19].Compound **4** was previously tested for DPPH, O_2_^−^·, OH and alkane free radical scavenging activity, and its IC_50_ values for eliminating the above free radicals were 39.5 µmol/L, 108.1 µmol/L, 172.5 µmol/L and 1832.4 µmol/L, respectively [26].Compound **6** was previously tested for DPPH and Fe^3+^reducing power ability and was a potent inhibitor of hydrogen peroxide (H_2_O_2_), its IC_50_ values for eliminating the above free radicals were 10.28 ± 0.24, 270.01 ± 7.37 and 316.43 ± 2.86 µmol/L, respectively [20]. Compound **7** was previously tested for DPPH radical scavenging activity with an IC_50_ = 18.2 ± 0.5 µmol/L [27]. Our results are similar to the literature.

The antioxidant activity of compound **7** is explained by the presence of the caffeoyl moities at C-3 and C-5 of the quinic acid, each having two phenol groups in the *ortho* position. The effective contribution of the two caffeoyl systems in compound **7** and even in its analogue **6** is confirmed by the considerable loss in the activity of compound **4** which contains only one in its structure.

The relative loss in antioxidant activity of compound **6** is certainly due to methylation of the carboxylic acid function which seems to contribute to this activity through its hydroxyl group. The significant antioxidant activity of compounds **6** and **7** may partly explain the use of this plant in traditional medicine against convulsions.

### 2.3. 5-Lipoxygenase Inhibitory

5-Lipoxygenase catalyzes the dioxygenation of polyunsaturated fatty acids to yield *cis, trans*-conjugated dienehydroperoxides. Results for 5-lipoxygenase inhibitory activity are shown in Table 3. Compound **6** showed a very good ability to inhibit 5-lipoxygenase (IC_50_ = 5.28 ± 0.1 µmol/L), better than NDGA positive standard (IC_50_ = 6.20 ± 0.5 µmol/L).Compound **7** has an interesting activity against 5-lipoxygenase, with an IC_50_ = 10 ± 0.2 µmol/L. The compounds **1** (16.9 ± 1.2%), **3** (12.4 ± 0.8%) and **4** (17.1 ± 1.1%) have moderate inhibition at 80 µmol/L. The other compounds **2**, **5**, **8**, **9**, **10** and **11** did not exhibit activities in this assay up to 80 µmol/L.

Compound **6** (IC_50_ = 5.28 ± 0.1 µmol/L) is twice as active as compound **7** (IC_50_ = 10 ± 0.2 µmol/L). This finding suggests the conclusion that the ester function has significantly improved the activity of compound **6** as compared to that of its analogue **7**. It is important to know that all products except **6** and **7** have never been tested for the anti-inflammatory(5-lipoxygenase) activity in the literature.The compounds **6** and **7** were tested against 5-lipoxygenase isolated from rat basophilic leukaemia cells (RBL-1). The compound **6** showed a low activity, with a percent inhibition of 15.1% at 20 µmol/L, while compound **7** was inactive at 20 µmol/L [28].

### 2.4. Cytotoxicity Evaluation

The cytotoxic activity of compounds **1**–**11** isolated from *F. lutea* flowers against the human colon carcinoma cell line HCT-116 and ovary cells lines IGROV-1 and OVCAR-3 was assessed using MTT assay, which is reliable to detect proliferation of cells. The activities of compounds were presented in Table 3. It is important to mention that none of these products have ever been previously tested for their cytotoxic activity against the human cell lines HCT-116, IGROV-1 and OVCAR-3.

Compounds **5** and **9** presented the best cytotoxic effect against the human colon carcinoma cell line HCT-116, with IC_50_ values of 22.5 ± 2.4 and 50.0 ± 4.9 µmol/L, respectively. The other compounds did not exhibit activities up to 100 µmol/L against the same cell line.

Only compound **5** exhibited the cytotoxic effects against the ovary cell lines IGROV-1 and OVCAR-3, with IC_50_ values of 17.8 ± 1.1 and 25 ± 1.1 µmol/L, respectively. The other compounds did not exhibit any activity up to 100 µmol/L against the IGROV-1 and OVCAR-3 cell lines.

Methyl caffeate (**5**) isolated from another plant, [29] was previously tested against human promyelocytic leukemia cell HL-60 (IC_50_ = 7.5 ± 1.6 µmol/L), human fibrosarcoma cell line HT-1080 (IC_50_ = 11.1 ± 3.5 µmol/L), colon cancer cell line LoVo (IC_50_ = 5.8 ± 1.1 µmol/L) and LoVo/Doxo (IC_50_ ≥ 40 µmol/L). Methyl 3,5-*O*-dicaffeoylquinate (**6**) was previously tested against liver cell line Hep-G2 and showed a moderate activity with an IC_50_ value of 72.7 ± 6.2 µmol/L [30].

Compound **5** showed higher toxicity against a colon cancer cell line (LoVo) and is less toxic against another type of colon cancer (HCT-116) and two ovary cancer cell lines (IGROV-1 and OVAR-3). Compound **6** showed a good activity against the liver cell line Hep-G2 but it showed no activity against HCT-116, OVCAR-3 and IGROV-1. This difference is due to the selectivity of the product towards the cancer cell lines.

The significant activity of caffeoyl acid methyl ester (**5**) towards the three cancer cell lines compared with that of its analogue where in caffeic acid is esterified with one of the alcohol functions of the quinic acid showed a considerable effect of the latter in reducing the activity of compound **5**. This conclusion is confirmed by the inactivity of compounds **6** and **7**, which both contain in their structures quinic acid and despite the presence of two caffeoyl moities.

The high activity of compound **9**, only against the HCT-116 cell line, might be explained by a selectivity phenomenon. The literature reports that narcissin **8** showed a low cytotoxic activity against the human chronic myelogenous leukemia cell line K562 with an IC_50_ value of 800 µmol/L [31].

## 3. Experimental Section

### 3.1. General Experimental Procedures

^1^H- (300 MHz), ^13^C- (75 MHz) and 2D-NMR spectra of the isolated compounds (except compound **8**) were recorded in CDCl_3_, CD_3_OD, acetone-d_6_ and DMSO-d_6_ with a Bruker NMR-300 spectrometer. ^1^H- (500 MHz), ^13^C- (125 MHz) and 2D-NMR spectra of compound **8** was recorded in DMSO-d_6_ with a Bruker NMR-500 spectrometer. The residual solvent resonances were used as the internal references. Coupling constants are given in Hertz. The chemical shifts are expressed in δ ppm. DCI-HRMS of compound **1** was run in a GCT 1^er^ Waters. ES-HRMS of compounds **2**, **6**, **7** and **8** were obtained by UPLC Xevo G2 Q TOF (Waters). DCI-MS of compounds **3**, **5**, **9** and **10** were obtained with a DSQ Thermo Fisher Scientific (DCI NH_3_). ES-MS of compound **4** was run in a Q-TRAP 2000 (Applied Biosystems). One µL of compound **11** dissolved in chloroform was injected in 1:10 split mode. Helium (purity 99.999%) was used as carrier gas at 1 mL/min. The injector was operated at 200 °C. The mass spectrometer (Varian Saturn GC/MS/MS 4D) was adjusted for an emission current of 10 μA and electron multiplier voltage between 1400 and 1500 V. Trap temperature was 220 °C and that of the transfer line was 250 °C. Mass scanning was from 40 to 650 amu. Crystal data for compound **2**, C_5_H_8_O_3_, were collected at 193K using a Bruker-APEX II Kappa Quazar Diffractometer, M = 116.11, monoclinic, *P2_1_/n*, a = 15.485 (5) Å, b = 4.7389 (15) Å, c = 15.967 (5) Å, β = 98.876 (15), V = 1157.7(6) A^3^, Z = 8, Dc = 1.332 mg/m^3^, X-ray source MoK α (radiation), λ = 0.71073 Å, F(000) = 496, colourless plate 0.20 × 0.08 × 0.04 mm. 11041 reflections were collected (1668 independent, Rint = 0.0609), 161 parameters. The structure solution was obtained by direct methods (SHELXS-97) and was refined with anisotropic thermal parameters using full-matrix least squares procedures on F^2^ to give R = 0.0751 using SHELXL-97 [32]. Cambridge Crystallographic Data Centre as supplementary publication number CCDC 1030232. Copies of the data can be obtained, free of charge, on application to CCDC, 12 Union Road, Cambridge CB2 1EZ, UK (fax: +44(0)-1223-336033 or e-mail: deposit@ccdc.cam.ac.uk).

### 3.2. Collection of Plant Material

*Ferula lutea* flowers were collected in the region of Béja (Tunisia), on April, 2010 and identified by Professor Féthia Harzallah Skhiri, in the Laboratory of Genetic, Biodiversity and Valorization of Bioresources, Higher Institute of Biotechnology of Monastir, University of Monastir, Tunisia. A voucher specimen was deposited in the same laboratory (F.L.F-10).

### 3.3. Extraction and Isolation 

The fresh flowers (5.8 kg) were macerated at room temperature with methanol/water (7/3, 20 L) for 7 days. The corresponding aqueous residue obtained after filtration and evaporation of the organic solvent (MeOH) under reduced pressure was partitioned successively with ethyl acetate and *n*-butanol yielding after evaporation of the solvents the corresponding ethyl acetate (56 g) and *n*-butanol (220 g) extracts. We used a CombiFlash^®^ Rf 200 flash chromatography system. This liquid chromatography apparatus allows us to split and purify several grams of an extract or a complex fraction with high throughput. It is performed using two pumps (5–200 mL/min; 0–200 psi), provided with packed columns (4 g to 330 g), a precolumn using RediSep Rf silica gel normal phase and a fraction collector with a diode array detector (200–780 nm).

The *n*-butanol extract (50 g) was further subjected to silica gel column chromatography eluting with petroleum ether/ethyl acetate (1000 mL × 90:10, 1000 mL × 70:30; 1000 mL × 50:50; 1000 mL × 30:70; 1000 mL × 10:90) and 1500 mL × ethyl acetate; ethyl acetate/methanol (1000 mL × 90:10; 70:30; 1000 mL × 50:50) and 1000 mL of methanol to afford 15 fractions. Fraction 5 (2.7 g) was fractioned by silica gel flash column chromatography using a gradient of chloroform/methanol (1500 mL × 90:10) and 800 mL of methanol to obtain compound **1** (45 mg). Fraction 4 (1.8 g) was fractioned by silica gel flash column chromatography using a gradient of chloroform/methanol (500 mL × 100:0; 800 mL × 80:20) and 500 mL × methanol to afford compound **2** (35 mg). The precipitation of the fraction 8 (3 g) in ethyl acetate gives an impure solid (1.1 g), which was fractioned by silica gel flash column chromatography using a gradient of chloroform/methanol (1200 mL × 80:20) to afford compound **3** (500 mg). Fraction 6 (2.7 g) was fractioned by silica gel flash column chromatography using gradient of chloroform/methanol (1000 mL × 90:10) and 600 mL of methanol, to yield compound **4** (45 mg). Fraction 1 (950 mg) was fractioned by silica gel flash column chromatography using gradient of dichloromethane/ethyl acetate (800 mL × 90:10) to obtain compound **5** (80 mg). 

The ethyl acetate extract (50 g) was subjected to silica gel column chromatography eluting with petroleum ether/ethyl acetate with increasing polarity (1000 mL × 100:0; 1200 mL × 90:10; 1500 mL × 80:20; 1500 mL × 60:40; 1500 mL × 50:50; 1500 mL × 30:70; 1200 mL × 0:100) then with 1000 mL × methanol to afford six fractions. Fraction 5 (8 g) was fractioned by silica gel flash column chromatography using gradient of cyclohexane/ethyl acetate (1800 mL × 50:50) and 500 mL of ethyl acetate to give six subfractions. The fifth subfraction was fractioned by silica gel flash column chromatography using gradient of cyclohexane/ethyl acetate (1000 mL × 30:70) and 300 mL of ethyl acetate to afford compounds **6** (320 mg) and **7** (40 mg). Fraction 6 (14 g) was further purified by silica gel column chromatography using gradient of chloroform/methanol (1300 mL × 70:30) to obtain compound **8** (67 mg). Fraction 4 (3 g) was fractioned by silica gel flash column chromatography using gradient of cyclohexane/ethyl acetate (1000 mL × 60:40) and 400 mL of ethyl acetate, to obtain 4 subfractions, the third of which was subjected to flash column chromatography using gradient of cyclohexane/ethyl acetate (800 mL × 60:40) to give compound **9** (15 mg). Fraction 2 (5.76 g) was fractioned by silica gel flash column chromatography using gradient of cyclohexane/ethyl acetate (600 mL × 90:10 and 800 mL × 80:20) and 300 mL of ethyl acetate, to obtain compound **10** (22 mg). The liquid layer of fraction 3 (9 g) when precipitated in methanol was fractioned by silica gel flash column chromatography using gradient of cyclohexane/ethyl acetate (700 mL × 90:10) and 500 mL × ethyl acetate to afford eight subfractions. The first subfraction was fractioned by silica gel flash column chromatography using gradient of cyclohexane/ethyl acetate (600 mL × 90:10) and 300 mL of ethyl acetate, to afford compound **11** (10 mg).

### 3.4. Spectroscopic and Physical Data of the Isolated Compounds 

*(E)-5-Ethylidenefuran-2(5H)-one-5-O-β-d-glucopyranoside* (ferunide, **1**): White amorphous powder; IR ν_max_ 3341, 1606 cm^−1^; DCI-HRMS: *m/z* 289.0916 [M+H]^+^; (Calcd for C_12_H_16_O_8_, 288.0845). For ^1^H and ^13^C-NMR spectroscopic data, see Table 2.

*(E)-4-Hydroxy-3-methylbut-2-enoic acid* (**2**): Colorless crystals; IR ν_max_3333, 1699 cm^−1^; ES-HRMS: *m/z* 115.0393 [M−H]^−^; (Calcd for C_5_H_7_O_3_, 116.0473). For ^1^H and ^13^C-NMR spectroscopic data, see Table 2.

*Verbenone-5-O-β-d-glucopyranoside* (**3**): White powder; ^1^H-NMR (CD_3_OD) δ 1.02 (3H, s, 9-CH_3_), 1.43 (3H, s, 8-CH_3_), 2.07 (3H, d, *J = * 1.5, 10-CH_3_), 2.46 (1H, d, *J = * 9.3, H-5_b_), 2.54 (1H, dd, *J*_1_ = 7.2; *J*_2_ = 2.7, H-6), 3.29 (1H, d, *J = * 9.3, H-5_a_), 3.12-3.41 (4H, m, H-2′, H-3′, H-4′, H-5′), 3.63 (1H, dd, *J*_1_ = 11.7; *J*_2_ = 5.1, H-6′_a_), 3.81 (1H, dd, *J*_1_ = 12; *J*_2_ = 2.1, H-6′_b_), 4.54 (1H, d, *J = * 7.5, H-1′), 5.66 (1H, q, *J = * 1.5, H-2). ^13^C-NMR (CD_3_OD) δ 205.4 (C-1), 177.1 (C-3), 121.5 (C-2), 100.2 (C-1′), 84.5 (C-4), 78.1 (C-3′), 77.8 (C-5′), 75.1 (C-2′), 71.7 (C-4′), 62.7 (C-6′), 61.7 (C-7), 52.8 (C-6), 44.7 (C-5), 23.0 (C-8), 20.5 (C-9), 20.3 (C-10). DCI-MS: *m/z* 329.2 [M+H]^+^, 327.2 [M−H]^−^.

*Chlorogenic acid* (**4**): White powder; ^1^H-NMR (CD_3_OD) δ 2.04 (2H, m, H-6), 2.15 (2H, m, H-2), 3.67 (1H, m, H-4), 4.10 (1H, m, H-5), 5.39 (1H, m, H-3), 6.29 (1H, d, *J = * 15.9, H-8′), 6.76 (1H, d, *J = * 8.1, H-5′), 6.93 (1H, dd, *J*_1_ = 8.4; *J*_2_ = 2.1, H-6′), 7.02 (1H, d, *J = * 2.1, H-2′), 7.57 (1H, d, *J = * 15.9, H-7′). ^13^C-NMR (CD_3_OD) δ 181.1 (C-7), 169.4 (C-9′), 149.8 (C-7′), 147.1 (C-3′), 147.1 (C-4′), 128.1 (C-1′), 123.2 (C-6′), 116.8 (C-2′), 115.9 (C-5′), 115.4 (C-8′), 78.0 (C-1), 75.3 (C-3), 73.3 (C-4), 72.9 (C-5), 40.9 (C-2), 39.3 (C-6). ES-MS: *m/z* 377.1 [M+Na]^+^.

*Methyl caffeate* (**5**): Green powder; ^1^H-NMR (acetone-d_6_) δ3.71 (3H, s, 10-CH_3_), 6.30 (1H, d, *J = * 15.9, H-8), 6.88 (1H, d, *J = * 8.1, H-5), 7.05 (1H, dd, *J*_1_ = 8.4; *J*_2_ = 2.1, H-6), 7.16 (1H, d, *J = * 2.1, H-2), 7.56 (1H, d, *J = * 15.9, H-7), 8.42 (OH). ^13^C-NMR (acetone-d_6_) δ168.0 (C-9′), 148.8 (C-7), 146.4 (C-3), 145.9 (C-4), 127.7 (C-1), 122.7 (C-6), 116.5 (C-2), 115.4 (C-5), 115.3 (C-8), 51.7 (C-10). DCI-MS: *m/z* 195.0 [M+H]^+^, *m/z* 212.0 [M+NH_4_]^+^.

*Methyl 3,5-O-dicaffeoylquinate* (**6**): yellow powder; ^1^H-NMR (CD_3_OD) δ2.18 (2H, m, H-6), 2.28 (2H, m, H-2), 3.66 (3H, s, 8-CH_3_), 3.96 (1H, m, H-4), 5.36 (1H, m, H-5), 5.44(1H, m, H-3), 6.21 (1H, d, *J = * 15.9, H-8′′), 6.33 (1H, d, *J = * 15.9, H-8′), 6.76 (1H, d, *J = * 8.1, H-5′′), 6.77 (1H, d, *J = * 8.1, H-5′), 6.92(1H, dd, *J*_1_ = 8.5; *J*_2_ = 1.8, H-6′′), 6.95(1H, dd, *J*_1_ = 8.5; *J*_2_ = 1.8, H-6′), 7.02 (1H, d, *J = * 1.8, H-2′′), 7.03 (1H, d, *J = * 1.8, H-2′), 7.54 (1H, d, *J = * 15.9, H-7′′), 7.61 (1H, d, *J = * 15.9, H-7′). ^13^C-NMR (CD_3_OD) δ176.2 (C-7), 169.3 (C-9′), 168.5 (C-9′′), 150.3 (C-7′), 150.1 (C-7′′), 148.0 (C-4′), 147.7 (C-4′′), 147.4 (C-3′), 147.3 (C-3′′), 128.4 (C-1′), 128.2 (C-1′′), 123.7 (C-6′), 123.6 (C-6′′), 117.2 (C-2′), 117.1 (C-2′′), 116.1 (C-5′), 116.0 (C-5′′), 115.7 (C-8′), 115.4 (C-8′′), 75.2 (C-4), 72.8 (C-3), 72.6(C-5), 70.3 (C-1), 53.6 (C-8), 37.6 (C-6), 37.3 (C-2). ES-HRMS: *m/z* 553.1324 [M+Na]^+^.

3*,5-O-dicaffeoylquinic acid* (**7**): White powder; ^1^H-NMR (CD_3_OD) δ2.21 (2H, m, H-6), 2.28 (2H, m, H-2), 3.97 (1H, m, H-4), 5.28 (1H, m, H-5), 5.35(1H, m, H-3), 6.34 (1H, d, *J = * 15.9, H-8′′), 6.35 (1H, d, *J = * 15.9, H-8′), 6.77 (1H, d, *J = * 8.1, H-5′′), 6.78 (1H, d, *J = * 8.1, H-5′), 7.01(1H, m, H-6′′), 7.01(1H, m , H-6′), 7.04 (1H, d, *J = * 1.8, H-2′′), 7.04 (1H, d, *J = * 1.8, H-2′), 7.55 (1H, d, *J = * 15.9, H-7′′), 7.58 (1H, d, *J = * 15.9, H-7′). ^13^C-NMR (CD_3_OD) δ177.9 (C-7), 169.4 (C-9′), 169.3 (C-9′′), 150.1 (C-7′), 150.1 (C-7′′), 148.0 (C-4′), 147.8 (C-4′′), 147.7 (C-3′), 147.6 (C-3′′), 128.5 (C-1′), 128.4 (C-1′′), 123.6 (C-6′), 123.6 (C-6′′), 117.7 (C-2′), 117.6 (C-2′′), 116.1 (C-5′), 116.0 (C-5′′), 115.8 (C-8′), 115.7 (C-8′′), 75.2 (C-1), 73.2 (C-3), 72.8(C-5), 72.6 (C-4), 37.4 (C-6), 35.9 (C-2). ES-HRMS: *m/z* 539.1168 [M+Na]^+^.

*Isorhamnetin-3-O-α-L-rhamnopyranosyl (1→6)-β-D-glucopyranoside* (narcissin, **8**): yellow powder; ^1^H-NMR (DMSO-d_6_) δ1.01 (3H, d,*J = * 6, 6′′′-CH_3_), 3.04-3.85 (8H, m, H-2′, H-3′, H-4′, H-5′, H-2′′′, H-3′′′, H-4′′′, H-5′′′), 3.83 (3H, s, 7′′-CH_3_), 4.92 (1H, d, *J = * 3.6, H-1′′′), 5.40 (1H, d, *J = * 7.5, H-1′′), 6.10 (1H, d, *J = * 1.5, H-8), 6.31 (1H, d, *J = * 1.5, H-6), 6.95 (1H, d, *J = * 8.4, H-5′), 7.52 (1H, dd, *J*_1_ = 8.4; *J*_2_ = 2.1, H-6′), 7.84 (1H, d, *J = * 1.8, H-2′). ^13^C-NMR (DMSO-d_6_) δ177.6 (C-4), 167.5 (C-7), 161.5 (C-5), 157.5 (C-2), 157.1 (C-9), 149.9 (C-3′), 147.6 (C-4′), 133.6 (C-3), 123.1 (C-6′), 121.9 (C-1′), 116.0 (C-5′), 113.8 (C-2′), 103.8 (C-10), 102.1(C-1′′′), 101.5 (C-1′′), 100.4 (C-8), 95.2 (C-6), 77.0 (C-5′′), 77.0 (C-3′′), 72.4 (C-4′′), 71.2 (C-3′′′), 70.9 (C-2′′′), 70.7 (C-4′′′), 69.0 (C-5′′′), 67.7 (C-6′′) 49.4 (C-7′), 18.1 (C-6′′′). ES-HRMS: *m/z* 647.1594 [M+Na]^+^.

*(−)-Marmesin* (**9**): White powder; ^1^H-NMR (CD_3_OD) δ1.13 (3H, s, 6′-CH_3_), 1.19 (3H, s, 5′-CH_3_), 3.25–3.13 (2H, m, H-3′_a_, H-3′_b_), 4.49 (1H, OH), 4.68 (1H, t, *J = * 9, H-2′), 6.10 (1H, d, *J = * 9.3, H-3), 6.62 (1H, s, H-8), 7.30 (1H, s, H-5), 7.76 (1H, d, *J = * 9.3, H-4). ^13^C-NMR (CD_3_OD) δ 163.9 (C-2), 162.4 (C-7), 155.5 (C-8_a_), 144.9 (C-4), 125.9 (C-6), 123.6 (C-5), 112.7 (C-4_a_), 110.7 (C-3), 96.8 (C-8), 91.1 (C-2′), 70.9 (C-4′), 29.4 (C-3′), 24.0 (C-5′), 23.9(C-6′). DCI-MS: *m/z* 247.1 [M+H]^+^.

*Isoimperatorin* (**10**): White powder; ^1^H-NMR (CDCl_3_) δ 1.69 (3H, s, 8′-CH_3_), 1.79 (3H, s, 7′-CH_3_), 4.92 (2H, d, *J = * 6.9, H-4′), 6.9(1H, m, H-5′), 6.28 (1H,d, *J = * 9.9, H-3), 6.95 (1H, d, *J = * 2.4, H-3′), 7.14 (1H, s, H-8), 7.59 (1H, d, *J = * 2.4, H-2′), 8.17 (1H, d, *J = * 9.9, H-4). ^13^C-NMR (CDCl_3_) δ 161.3 (C-2), 158.1 (C-7), 152.7 (C-8_a_), 149.0 (C-5), 144.9 (C-2′), 139.8 (C-6′), 139.6 (C-4), 119.1 (C-5′), 114.2 (C-6), 112.6 (C-4_a_), 107.5 (C-3), 105.1 (C-3′), 94.2 (C-8), 69.8(C-4′), 25.8 (C-7′), 18.2 (C-8′). DCI-MS: *m/z* 271.0 [M+H]^+^, 288.1 [M+NH_4_]^+^.

*2,3,6-Trimethylbenzaldehyde* (**11**): White powder; ^1^H-NMR (CDCl_3_) δ 2.17 (3H, s, 3′-CH_3_), 2.35 (3H, s, 2′-CH_3_), 2.60 (3H, s, 4′-CH_3_), 7.17 (1H, d, *J = * 7.8, H-5), 7.57 (1H, d, *J = * 7.8, H-4), 10.25 (1H, s, H-1′). ^13^C-NMR (CDCl_3_) δ 193.1 (C-1′), 143.2 (C-6), 139.0 (C-2), 136.7 (C-3), 132.6 (C-1), 129.6 (C-4), 127.7 (C-5), 21.6 (C-4′_)_, 15.3 (C-2′), 14.8 (C-3′). GC-MS: *m/z* 148.0 [M]^+^.

### 3.5. Biological Activity

The absorptance of the extract, at the wavelength of each test and with each concentration, was removed to eliminate the effect of colouring.

#### 3.5.1. Free Radical Scavenging Activity DPPH Test

The antioxidant activity of compounds was assessed according to their DPPH scavenging ability as described by Khlifi *et al.*, [33] with some modifications. Reaction mixtures, containing 0.1 mL of the relevant compounds solution in methanol, with concentrations ranging 3 mg/mL and 0.1 mL of a 0.2 mM DPPH methanolic solution, were added to 96-well microtiter plates and incubated at 25 °C for 30 min. Absorbances were measured at 520 nm, the wavelength of maximum absorbance of DPPH, were recorded as A_(sample)_. A blank experiment was also carried out applying the same procedure to a solution without the test material and the absorbance was recorded as A_(blank)_. The free radical-scavenging activity of each solution was then calculated as percent inhibition according to the following equation:

% inhibition = 100 × (A _(blank)_ − A_(sample)_)/A_(blank)_) (1)

Antioxidant activity extracts was expressed as IC_50_, defined as the concentration of the test material required to cause a 50% decrease in initial DPPH concentration. Ascorbic acid was used as a standard. All measurements were performed in triplicate.

#### 3.5.2. ABTS Radical-Scavenging Test

The radical scavenging capacity of the samples for the ABTS (2,2′-azinobis-3-ethylbenzothiazoline-6-sulphonate) radical cation was determined as described by Khlifi *et al.*, [33] with some modifications. ABTS was generated by mixing a 7 mM of ABTS at pH 7.4 (5 mM NaH_2_PO_4_, 5 mM Na_2_HPO_4_ and 154 mM NaCl) with 2.5 mM potassium persulfate (final concentration) followed by storage in the dark at room temperature for 16 h before use. The mixture was diluted with ethanol to give an absorbance of 0.70 ± 0.02 units at 734 nm using spectrophotometer. For each sample, diluted solution (20 μL) was allowed to react with fresh ABTS solution (180 μL), and then the absorbance was measured 6 min after initial mixing. Ascorbic acid was used as a standard and the capacity of free radical scavenging was expressed by IC_50_ (µmol/L) values calculated denote the concentration required to scavenge 50% of ABTS radicals. The capacity of free radical scavenging IC_50_ was determined using the same previously used equation for the DPPH method. All measurements were performed in triplicate.

#### 3.5.3. 5-Lipoxygenase Inhibitory

The anti-inflammatory activity activities of the isolates was determined on Soybean lipoxygenase as described by Khlifi *et al.* [33] with some modifications. 20 µL of each compound was mixed individually with 150 µL sodium phosphate buffer (pH 7.4) containing 20 µL of 5-lipoxygenase and 60 µL of linoleic acid (3.5 mM), yielding a final volume of 250 µL. However, the blank does not contain the substrate, but will be added 30 µL of buffer solution. All compounds were re-suspended in the DMSO followed by dilution in the buffer so that the DMSO does not exceed 1%. The mixture was incubated at 25 °C for 10 min, and the absorbance was determined at 234 nm. The absorption change with the conversion of linoleic acid to 13-hydroperoxyoctadeca-9,11-dienoate (characterized by the appearance of the conjugated diene at 234 nm) was followed for 10 min at 25 °C. Nordihydroguaiaretic acid (NDGA) was used as positive control. The percentages of enzyme activity were obtained at 200 µmol/L. All measurements were performed in triplicate.

#### 3.5.4. Cytotoxicity Evaluation 

The human cancer cell line was used for cytotoxic assay: (HCT-116, IGROV-1 and OVCAR-3). The cells were grown in RPMI-1640 medium supplemented with 10% faetal calf serum (Gibco, Langley, OK, USA), air and 5% CO_2_. The isolate was added to a medium containing 1 × 10^6^ cells/mL, 2 mM l-glutamine and 50 μg/mL gentamycin, and kept at 37 °C in a fully humidified atmosphere. After 18 h of incubation at 37 °C in 5% CO_2_ incubator, the tubes were centrifuged at 8000 *g* for 10 min. The supernatant was decanted, and the pellets taken and washed with 20 mM of phosphate buffered saline solution. Each pellet was dissolved in 100 μL (2 mg/mL) MTT solution in a tube, incubated at 22 °C for 4 h and centrifuged at 8000 *g* for 10 min. All the pellets were dissolved in 500 μL DMSO and read spectrophotometrically at 500 nm. IC_50_ was calculated by nonlinear regression analysis. Doxorubicin (HCT116) and tamoxifen (IGROV-1 and OVCAR-3) were used as positive control.

## 4. Conclusions

Two new natural compounds: ethylidenefuran-1-one-5-*O*-β-d-glucopyranoside (**1**) and 4-hydroxy-3-methylbut-2-enoic acid (**2**) were isolated together with verbenone-5-*O*-β-d-glucopyranoside (**3**), chlorogenic acid (**4**), methyl caffeate (**5**), methyl 3,5-*O*-dicaffeoylquinate (**6**),3,5-*O*-dicaffeoylquinic acid (**7**) isorhamnetin-3-*O*-α-l-rhamnopyranosyl(1→6)-β-d-glucopyranoside (narcissin **8**), (−)-marmesin (**9**), isoimperatorin (**10**) and 2,3,6-trimethylbenzaldehyde (**11**) from the flowers of *F. lutea*. Their structures were elucidated by spectroscopic methods including 1D, 2D-NMR experiments, mass spectroscopic and X-ray diffraction for compound **2**. Antioxidant, anti-inflammatory and cytotoxic activities were evaluated. Compound **7** exhibited the higher antioxidant activity. Compound **6** showed the higher activity against 5-lipoxygenase compared to NDGA. Compound **5** was also found to be cytotoxic against the HCT 116, IGROV-1 and OVCAR-3 cell lines and compound **9** is cytotoxic specifically against the HCT 116.
